# Acute neuromuscular fatigue in circuit strength training: effects of varying work-to-rest durations

**DOI:** 10.3389/fspor.2026.1761646

**Published:** 2026-02-04

**Authors:** Aaron Agudo-Ortega, Sara Echeberria-Castaño, Javier Iglesias García, Adrián Martín-Castellanos, Juan Ramón Heredia-Elvar, Francisco Hermosilla-Perona

**Affiliations:** 1 AExPH, Facultad de Ciencias Biomédicas y de la Salud, Universidad Alfonso X el Sabio (UAX), Avenida de la Universidad, Madrid, España; 2 Facultad de Ciencias de la Salud, Universidad Internacional de La Rioja. Avenida de la Paz, La Rioja, España; 3 Facultad de Ciencias de la Vida y la Naturaleza, Universidad Nebrija, Madrid, Spain

**Keywords:** acute effects, circuit training, fatigue, neuromuscular performance, strength

## Abstract

**Introduction:**

Circuit training (CT) is a time-efficient resistance training method widely used to elicit neuromuscular and metabolic adaptations. However, the influence of different work-to-rest interval durations within a fixed 1:1 ratio on acute neuromuscular fatigue remains unclear. The present study aims to examine the acute effects of different 1:1 CT work-to-rest interval durations (10s, 20s, and 30s) on neuromuscular fatigue.

**Methods:**

Thirty-four physically active participants (15 males and 19 females) completed 14-min CT protocols, each consisting of two 6-min blocks separated by a 2-min rest period and involving six alternating upper- and lower-body exercises (leg press, bench press, unilateral right knee extension, bilateral row, unilateral left knee extension, and seated overhead press). CT includes six exercises alternating lower- and upper-body movements. Mean propulsive velocity (MPV) was measured for squat (SQ) and bench press (BP) exercises before and after each session, and the total number of repetitions performed per exercise and set was recorded.

**Results:**

A significant main effect of time (pre- vs. postdata) was observed for MPV in both the SQ (*p* < 0.001) and the BP (*p* < 0.001) exercises. Velocity loss revealed significant differences between the SQ and the BP exercises (*p* < 0.001). The 30:30 protocol consistently performed a higher repetition rate (0,612–0,806 rep/s) compared with both 10:10 (0,488–0,768 rep/s) and 20:20 (0,488–0,754 rep/s) protocols.

**Conclusions:**

A significant reduction in MPV was observed following all protocols, indicating the presence of acute neuromuscular fatigue regardless of interval duration. Although no statistically significant differences in fatigue magnitude were detected between protocols, work-to-rest interval duration influenced repetition output and execution characteristics, with the 30:30 protocol allowing greater repetition volume compared with the 10:10 and 20:20 protocols.

## 1 Introduction

Circuit Training (CT) conventionally consists of approximately 10–15 resistance exercises targeting different muscle groups, each performed for 12–15 repetitions at 40%–60% of one-repetition maximum (1RM), completed in 30–40 s (s) per station with 15–30 s rest between stations; the circuit is usually repeated one to three times for a total session of approximately 30 min (1, 2). Owing to this time-efficient structure, CT has garnered increasing scientific interest and has been shown to produce concurrent gains in muscular endurance, aerobic capacity, strength, and power, while requiring roughly 66% of the time demanded by traditional resistance methods (3–5).

Training management involves variables such as intensity, volume, frequency, exercise sequence, and work-to-rest ratio. In this context, intensity is determined by the maximal velocity performed during the first repetition (6, 7). In terms of volume, training is frequently prescribed through the pre-establishment of a fixed number of repetitions to be completed per exercise (8). This approach is commonly operationalized through the XRM concept, which defines relative intensity based on the exact number of repetitions performed (3, 4, 9, 10). However, in the specific context of CT, where work periods are strictly time-constrained rather than repetition-constrained, this repetition-based prescription may be particularly problematic. When exercise duration is limited by time, the actual number of repetitions completed can vary substantially between sets and individuals because of fatigue, pacing strategies, and recovery availability. As a result, the intended relative intensity and training stimulus associated with a given XRM prescription may not be consistently achieved across a circuit-based session. Conventionally, it has been assumed that performing the maximal number of repetitions with a previously prescribed load is indicative of a constant percentage of relative intensity. Nevertheless, this assumption has been challenged, as within the range of 50%–85% of 1RM. Both the perceived exertion and the resultant physiological adaptations may exhibit variability of up to 20% (6, 8). Hence, the integration of training variables in CT is crucial for optimizing neuromuscular, cardiorespiratory, and body composition adaptations that collectively determine the effectiveness of these protocols (9). Consequently, when training load and volume are prescribed using approaches that may not accurately reflect the actual mechanical and physiological demands imposed during time-constrained exercise, such as CT, there is an increased risk of unintended variability in the training stimulus. Under these conditions, neuromuscular fatigue emerges as a central, inherently complex, multifactorial, and highly task-dependent phenomenon (11, 12) that is characterized by a diminishment in the ability to produce muscular force or power (13).

While the interaction between intensity and volume has traditionally been posited as a principal mechanism underlying fatigue onset (10), recent advances have identified velocity loss as a robust, objective, and non-invasive indicator of neuromuscular fatigue during resistance training. In this context, previous research (14) demonstrated that reductions in movement velocity within a set closely reflect the degree of neuromuscular fatigue, providing a practical framework for monitoring fatigue and training load beyond traditional repetition-based approaches. Beyond training, the monitoring of movement velocity provides real-time feedback on acute metabolic stress, endocrine responses, and mechanical fatigue experienced within a session (15).

Furthermore, the temporal structure is a key determinant of the physiological and neuromuscular demands elicited by circuit training protocols (16, 17). In this regard, training formats closely related to CT, such as high-intensity interval training (HIIT), have demonstrated that the manipulation of work-to-rest intervals critically influences metabolic stress, neuromuscular fatigue, and performance sustainability (13, 18). These principles are directly applicable to CT, where rest intervals between exercises modulate both the immediate physiological response and the accumulation of fatigue across the session. Between sets, rest-interval duration is considered a key factor for maintaining a consistent repetition count and for modulating specific training adaptations (19). To enhance muscular endurance, extremely brief rest intervals (e.g., 30 s) force a reduction in intensity in subsequent sets to keep repetitions within the prescribed range. Even at relatively low loads (40%–60% 1RM), the use of very short rests (<1 min) does not always allow maintenance of repetition count per set (20, 21).

It has also been postulated that the duration of a rest interval might depend on the duration of work during the set (22). Although empirical evidence increasingly supports the effectiveness of 1:1 and 2:1 work-to-rest ratios, there is still no consensus regarding a definitive standard in the scientific community (9). Moreover, it has been evidenced that prolonged work intervals combined with minimal rest periods exacerbate the decline in neuromuscular performance (4, 9, 23, 24). To our knowledge, scientific literature has addressed specific 1:1 configurations (e.g., 30:30 and 15:15 s) in circuit training protocols, integrating non-uniform session durations and distinct exercise counts (25). Within this framework, it becomes fundamental to thoroughly elucidate the influence of divergent work-to-rest configurations on the acute modulation of neuromuscular fatigue. Therefore, the present study aimed to analyze the acute effects of three different circuit training protocols (10:10, 20:20, and 30:30 s), all characterized by a 1:1 work-to-rest ratio, on neuromuscular fatigue. It was hypothesized that all protocols would induce significant prepost reductions in neuromuscular performance, as reflected by decreases in mean propulsive velocity. In addition, it was hypothesized that longer work-to-rest intervals would allow greater repetition output and better maintenance of execution characteristics, despite similar magnitudes of neuromuscular fatigue across protocols.

## 2 Materials and methods

### 2.1 Participants

Thirty-four adult healthy individuals (15 males and 19 females) within the age range of 19–35 years voluntarily agreed to participate in the study. The sample size was calculated using G*Power software (version 3.1.9.2, Kiel, Germany) based on a repeated-measures analyses of variance (ANOVA) design. The analysis assumed an alpha level of 0.05, a statistical power of 80% (1−*β*), and a large effect size (*f* = 0.5). This effect size was selected based on previous acute resistance training studies reporting large prepost decrements in neuromuscular performance variables following high-intensity circuit or intermittent strength training protocols. A moderate correlation among repeated measures (*r* = 0.5) was assumed, consistent with prior velocity-based resistance training research. Under these assumptions, a minimum sample size of 30 participants was required. Only individuals with a minimum of six months of continuous resistance training experience and no injuries or musculoskeletal disorders during this period were included in the study. Consistent training practice was defined as engaging in resistance training at least three times per week. Body mass and height were not recorded, as these variables were not required for the objectives of the present study and did not directly influence the velocity-based neuromuscular outcomes assessed. As stipulated by McKay et al. (26), our participants were classified as Tier 1. Furthermore, participants were instructed to maintain their regular dietary habits as well as to refrain from performing intense physical activity for 48 h approximately before each training session. The study was conducted in accordance with the Declaration of Helsinki and was approved by the Bioethics Committee of the Alfonso X El Sabio University (approval code: 2024_12/312). All participants provided written informed consent prior to participation.

### 2.2 Experimental design

A cross-sectional experimental study was conducted. The identical cohort of 34 participants underwent the same training protocols that comprised the study. The implementation of three circuit-based resistance training protocols was involved, each one with a fixed total duration of 14 min. Nevertheless, each protocol differed in terms of work-to-rest intervals: 10:10 (10 s of work, 10 s of rest); 20:20 (20 s of work, 20 s of rest); and 30:30 (30 s of work, 30 s of rest).

The participants completed three separate sessions, each separated by a minimum intersession recovery period of 48 h. All testing sessions were scheduled at the same time of day for each participant to control for potential time-of-day effects on neuromuscular performance. The study was conducted over a 4-week data collection period. The order in which the three circuit training protocols were administered was randomized and counterbalanced across participants to minimize potential carryover and learning effects inherent to repeated-measures designs. Each 14-min session was divided into two 6-min blocks separated by a 2-min active rest period. All testing sessions were conducted in Villanueva de la Cañada (Madrid, Spain; approximately 650 m above sea level). The sessions took place in a climate-controlled gym environment, with ambient temperature maintained at approximately 20°C–22 °C.

### 2.3 Procedures

One week before the data collection phase began, a familiarization session was conducted to familiarize the participants with the circuit training structure, exercise sequence, and velocity-based measurement procedures, as well as to determine individualized training loads for each protocol. During this session, the participants were instructed to perform as many repetitions as possible at maximal intended velocity within each work interval to determine the load lifted at muscle failure. This procedure was repeated for each station in the circuit. During the experimental protocol sessions, the load used at each station was set at one plate less than that employed during the familiarization session. This approach ensured ecological validity and allowed the participants to complete the protocols with a high level of effort without reaching muscular failure. Notably, shorter work-to-rest ratios required higher absolute training loads (e.g., 10:10 > 20:20 > 30:30) to achieve comparable task demands.

All participants completed the same standardized warm-up protocol prior to each testing session. The warm-up consisted of an 8-min treadmill run at a self-selected brisk walking speed, followed by ten repetitions each of shoulder flexion-extension, abduction-adduction, and circumduction exercises. The participants then performed eight progressive bodyweight squats. Finally, they completed one familiarization lap of the circuit, performing six repetitions at each station with 50% of the prescribed training load for the intermediate protocol (20:20), with 30 s of rest between exercises.

**Figure 1:**
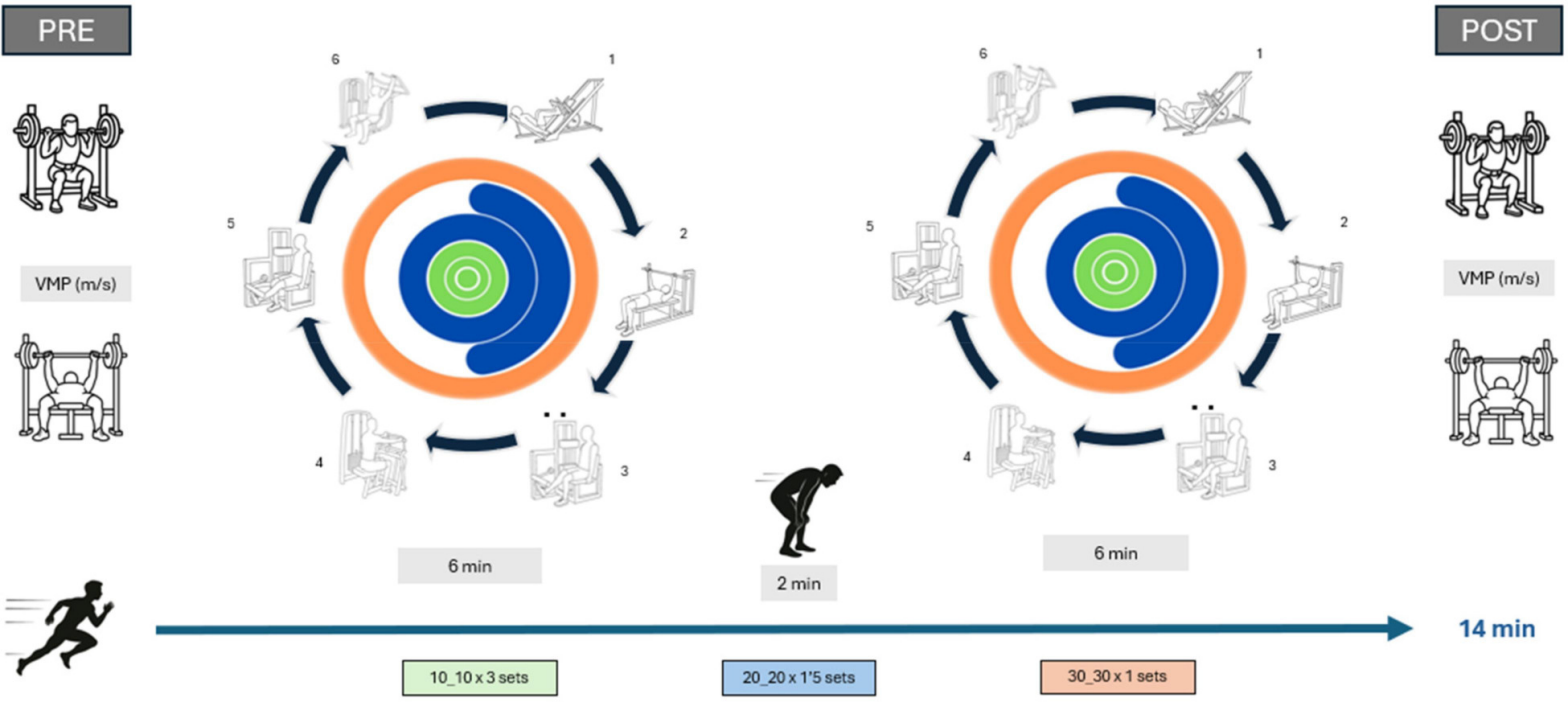
Circuit training data collection.

The two blocks of CT consisted of six machine-based exercise stations performed in the following sequence: (1) leg press (bilateral lower-body pushing exercise targeting the knee and hip extensors), (2) bench press (bilateral upper-body horizontal pushing exercise primarily involving the pectoralis major, anterior deltoid, and triceps brachii), (3) unilateral knee extension (right leg; single-joint knee extensor exercise), (4) bilateral seated row (upper-body horizontal pulling exercise targeting the latissimus dorsi and upper back musculature), (5) unilateral knee extension (left leg; single-joint knee extensor exercise), and (6) seated overhead press (bilateral upper-body vertical pushing exercise involving the deltoids and triceps brachii). All exercises were performed using BH Fitness equipment (BH Fitness, Vitoria, Spain). After and before each protocol (Figure 1), Mean propulsive velocity (MPV) was collected within two traditional strength exercises, bench press (BP) and squat (SQ). MPV was selected for its sensitivity in reflecting the load–velocity relationship at moderate intensities (∼45% 1RM for bench press and ∼60% 1RM for squat) and for its strong participant tolerance (12). Repetitions were systematically recorded during each designated work period. Verbal encouragement was constantly provided to enhance maximal effort (12).

To ensure consistency and reliability, posture and load were replicated across sessions. A progressive loading protocol was employed, beginning with unloaded barbell movements as a specific warm-up. Thereafter, the participants performed successive sets with increasing absolute loads. Initial loads were set at low intensities and subsequently increased in a stepwise manner, with load increments informed by the individual load–velocity data obtained during the familiarization period. At each load, two repetitions were performed, and the repetition with the highest velocity was retained for analysis. Load progression continued until an MPV of approximately 1 m/s was achieved. The target MPV was considered reached when the highest velocity repetition at a given load was closest to 1 m/s, and this load was used for both pre- and posttest measurements (14, 27). The participants were instructed to avoid bouncing the bar and to maintain correct technique throughout both eccentric and concentric phases. For each load, two repetitions were performed and the repetition with the highest velocity was chosen for analysis. MPV was measured using the Vitruve Encoder (Speed4Lift, Madrid, Spain). Along with these measurements, the rating of perceived exertion (RPE) was assessed using the Borg CR-10 scale. RPE data were collected twice, immediately after the first 6-min exercise block (during the 2-min rest period) and immediately after the completion of the entire 14-min circuit.

### 2.4 Statistical analysis

In this study, the assumption of normality was verified using the Shapiro–Wilk test, while the assumption of homogeneity of variances (homoscedasticity) was assessed using Levene's test (*p* > 0.05). For repeated-measures analyses, the assumption of sphericity was evaluated using Mauchly's test. When violations of sphericity were detected, Greenhouse–Geisser corrections were applied to adjust the degrees of freedom. To assess changes in MPV, repeated-measures ANOVA were performed separately for the BP and the SQ exercises. Each model included time (pre, post) and protocol (10:10, 20:20, and 30:30) as within-subject factors. The main effects of time and protocol, as well as the time * protocol interaction, were examined. When appropriate, Bonferroni-adjusted *post hoc* comparisons were performed to identify specific differences between time points (pre *vs*. post) following significant main effects of time, and between protocols (10:10 vs. 20:20, 10:10 vs. 30:30, and 20:20 vs. 30:30) following significant main effects of protocol or time * protocol interactions. To compare velocity loss between exercises and protocols, a mixed-design ANOVA was conducted, with exercise (SQ *vs*. BP) as a within-subject factor and protocol (10:10, 20:20, and 30:30) as a between-subject factor. Effect sizes for pairwise comparisons derived from paired-samples *t*-tests were calculated using Cohen's d (1988) and interpreted as small (*d* ≈ 0.20), medium (*d* ≈ 0.50), or large (*d* ≥ 0.80). Effect sizes for repeated-measures ANOVA were reported separately as partial eta squared (*η*^2^). The number of repetitions per exercise throughout the circuit was reported in absolutes values. To allow for comparisons, the values were standardized to “repetitions per second” to compare the different exercises across the three protocols. RPE was collected to provide complementary information on perceived effort during the protocols but was not included in the inferential statistical analyses, as it was not directly related to the primary neuromuscular outcomes of the study. The statistical analysis was conducted using JASP software (version 0.19.3.0).

## 3 Results

With regard to sex differences, although males generally exhibit greater absolute strength levels, tendencies toward fatigue and velocity loss when completing the same circuit training protocols were similar regardless of sex. To verify this, an ANOVA including sex (males vs. females) and protocol (10:10, 20:20, and 30:30) as factors was performed for both bench press and squat performance. No significant main effect of sex was observed for BP (*F* = 1.621, *p* = 0.206) or SQ (*F* = 1.641, *p* = 0.203), and no significant sex×protocol interactions were found for BP (*F* = 0.843, *p* = 0.434) or SQ (*F* = 0.513, *p* = 0.600). Consequently, data from male and female participants were pooled and the results are presented jointly.

A significant main effect of time (“Moment”) was observed for MPV in both the SQ (*F* = 11.75, *p* < 0.001, *η*^2^ = 0.162) and the BP (*F* = 276.27, *p* < 0.001, *η*^2^ = 0.234) exercises, indicating a clear reduction in movement velocity after the CT protocols. No significant main effect of “Group” for SQ (*F* = 1.098, *p* = 0.338, *η*^2^ = 0.015) and BP (*F* = 0.014, *p* = 0.986, *η*^2^ = 1.928*10^−4^) or of “Moment * Group” for SQ (*F* = 2.508, *p* = 0.087, *η*^2^ = 0.007) or BP (*F* = 0.268, *p* = 0.765, *η*^2^ = 4.542*10^−4^) interactions was detected. In terms of velocity loss (VL), no significant differences were found between protocols for either the SQ (*F* = 2.822, *p* = 0.064, *η*^2^ = 0.054) or the BP (*F* = 0.100, *p* = 0.905, *η*^2^ = 0.002) exercises. These results are summarized in Table 1.

**Table 1:** ANOVA interaction effect variables.

Variables		Df	*F*	*p*	*η* ^2^
MPV squat	Time (pre vs. post)	1	11.751	<0.001	0.162
	Time * Protocol	2	2.508	0.087	0.007
	Protocol	2	1.098	0.338	0.015
MPV bench press	Time (pre vs. post)	1	276.274	<0.001	0.234
	Time * Protocol	2	0.268	0.765	4.542×10^−4^
	Protocol	2	0.014	0.986	1.928×10^−4^
VL squat	Protocol	2	2.822	0.064	0.054
VL bench press	Protocol	2	0.100	0.905	0.002

MPV, mean propulsive velocity; VL, velocity loss; *F*, Fisher's F ratio; Df, degrees of freedom; *η*^2^, partial eta squared; *p*, probability value.

**Figure 2:**
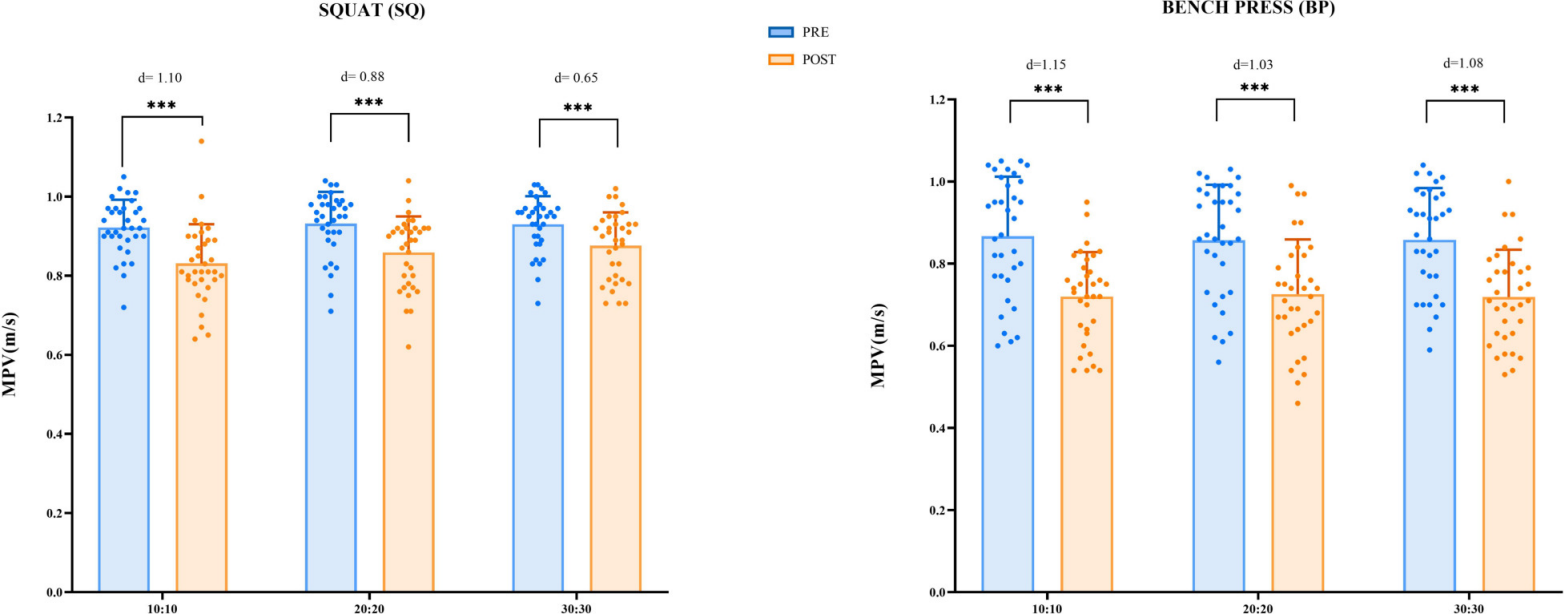
Mean propulsive velocity in squat and bench press. Absolute participant values (plots) and Cohen's effect size (in text) are presented.

The results of MPV are presented in Figure 2. With regard to the Squat (SQ) exercise, a highly significant main effect of time (pre- and postdata) on MPV was observed (*p* < 0.001). *Post hoc* comparisons revealed significant differences in MPV from pre- to postdata across all protocols proposed: 10:10 (*p* < 0.001); 20:20 (*p* < 0.001); 30:30 (*p* < 0.001). Effect sizes ranged from moderate to large across conditions (30:30 < 20:20 < 10:10). The interaction between time at the different protocols proposed was not significant (*p* < 0.09). Similarly, in the BP exercise, a significant main effect on time was observed (*p* < 0.001). *Post hoc* comparisons indicated significant differences between pre- and postdata measurements in all conditions: 10:10 (*p* < 0.001); 20:20 (*p* < 0.001); 30:30 (*p* < 0.001). Effect size values were large in all conditions (>0.8). The interaction between time at the different protocols proposed was not significant (*p* = 0.765). No significant within-subject differences were found (*p* = 0.986).

**Figure 3:**
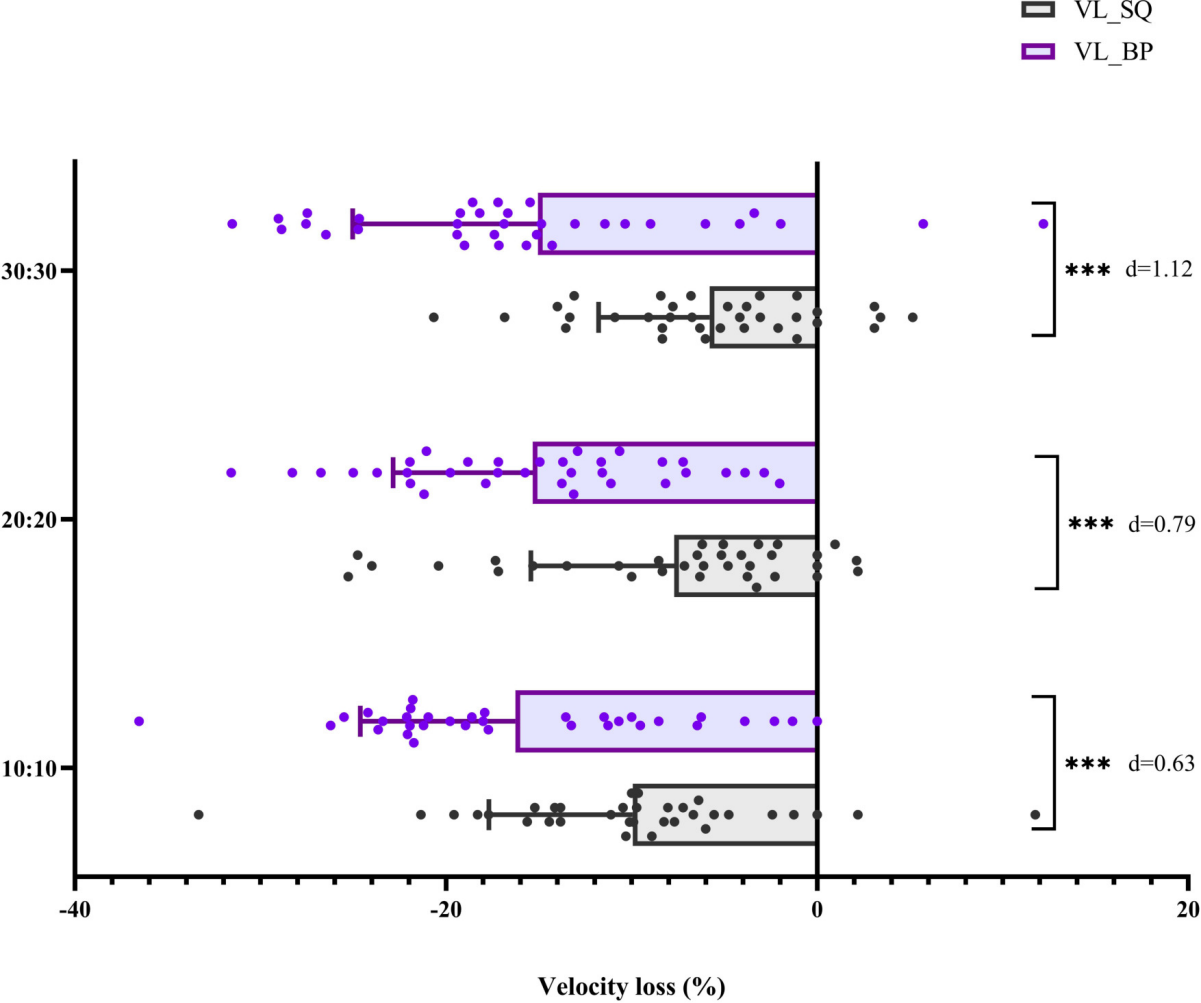
Velocity loss in squat and bench press. Absolute participant values (plots) and Cohen's effect size (in text) are presented.

With regard to velocity loss (Figure 3), the mixed-model ANOVA revealed a significant main effect of exercise, with greater velocity loss observed in the BP exercise compared with the SQ exercise (*F* = 71.34, *p* < 0.001, *η*p^2^ = 0.419). No significant main effect of protocol was found (*F* = 1.19, *p* = 0.310, *η*p^2^ = 0.023), indicating that velocity loss did not differ between the 10:10, 20:20, and 30:30 circuits. In addition, no significant exercise * protocol interaction was observed (*F* = 1.29, *p* = 0.281, *η*p^2^ = 0.025).

**Figure 4:**
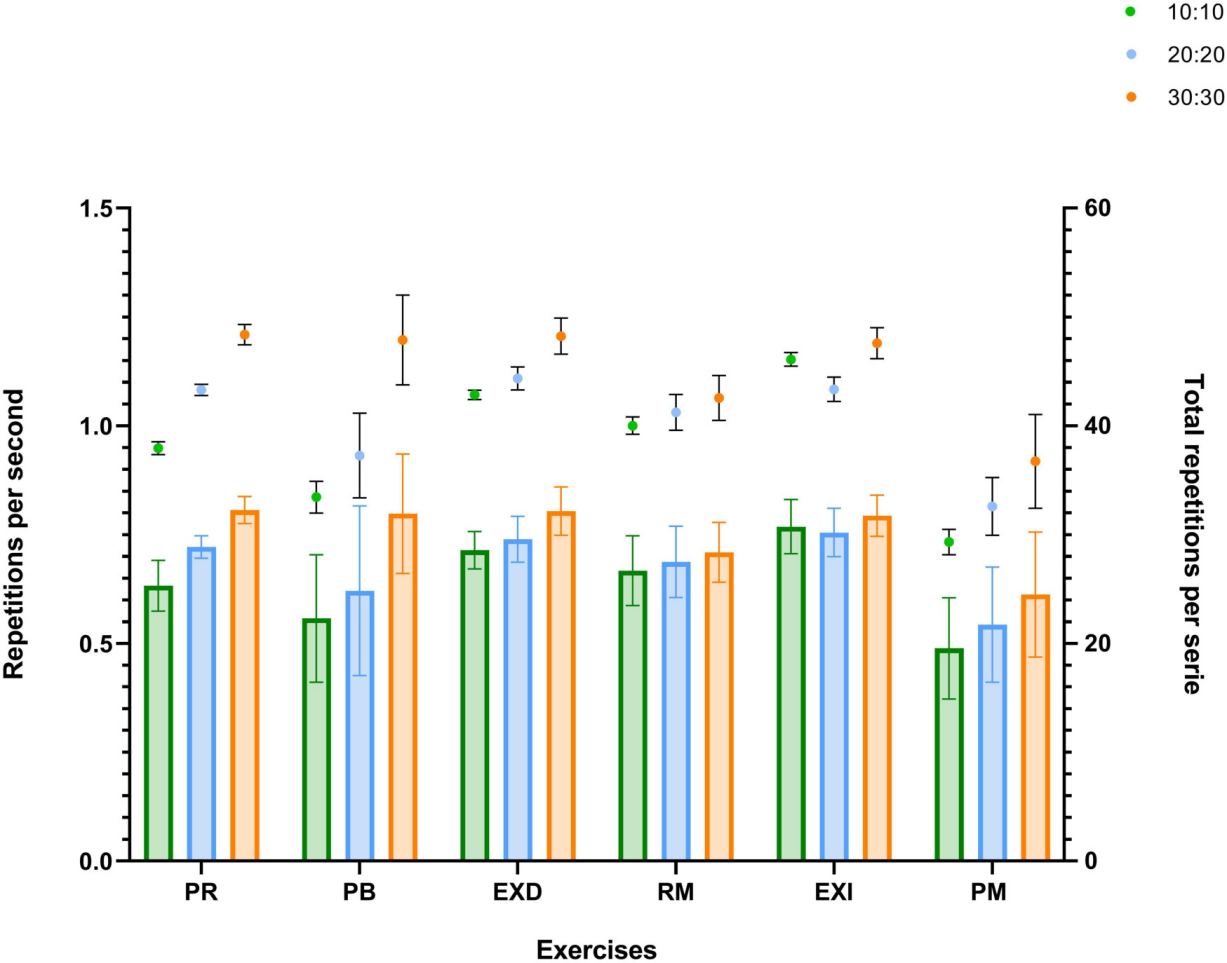
Repetitions per second per protocol (left *y*-axis) are presented in bars and total repetitions per set (right *y*-axis) in plots are displayed. PSS, leg press; BP, bench press; REXT, right knee extension; RO, row; LEXT, left knee extension; OPSS, overhead press.

Figure 4 illustrates the number of repetitions performed per second (left *y*-axis), together with the total repetitions completed per set (right *y*-axis). It can be observed that the 30:30 group consistently performed a greater number of repetitions (0.612–0.806 rep/s) compared with the 10:10 (0.488–0.768 rep/s) and 20:20 (0.488–0.754 rep/s) protocols, both in terms of repetitions per second and in terms of total repetitions per set.

## 4 Discussion

This study examined the acute effects of three different CT protocols on neuromuscular fatigue with a 1:1 work-to-rest ratio but which differed in terms of the duration of each interval (10:10, 20:20, and 30:30). Significant differences were found in MPV in the SQ and BP exercises between pre- and postdata but not between protocols. In terms of velocity loss, significant differences were found between the SQ and the BP exercises without significant differences between the distinct time intervals. Although no statistically significant differences were observed between protocols, the 10:10 condition tended to show a greater decline in performance without reaching statistical significance compared with the 20:20 and 30:30 protocols, suggesting a potentially higher fatigue demand under very short work-to-rest intervals. Finally, the number of repetitions revealed that the 30:30 protocol enabled a greater total number of repetitions per second and total repetitions per set in comparison with the 10:10 and 20:20 protocols.

MPV was recorded under the same absolute load at both pre- and posttest for the BP and the SQ exercises. Significant differences between these two moments across each work-recovery interval confirmed the presence of training-induced fatigue, which is a widely recognized reliable indicator of neuromuscular fatigue (8, 12). Moreover, MPV values descriptively tended to be lower following the 10:10 protocol compared with the 30:30 condition, although these differences did not reach statistical significance. This result indicated that participants experienced greater differences when the rest interval was limited to only 10 s (28).

With regard to the percentage of velocity loss, significant differences were assessed between BP and SQ, supporting previous evidence that exercises involving larger muscle groups induce greater fatigue (29). Certainly, the capacity to sustain the maximal number of repetitions across multiple sets appears to be less efficient in upper-body exercises compared with lower-body exercises (29, 30). In contrast, no statistically significant differences were found between the different circuit configurations, although descriptive data indicated a tendency toward greater velocity loss in the 10:10 protocol. This trend supports the notion that very short work-to-rest intervals may impose higher metabolic stress and contribute to greater peripheral fatigue compared with longer intervals and therefore should be interpreted as a practical tendency rather than a statistically confirmed effect (27). From a physiological perspective, reducing the absolute duration of work-to-rest intervals may exacerbate peripheral fatigue because of insufficient recovery of intramuscular homeostasis between successive bouts. Very short recovery periods limit phosphocreatine resynthesis and increase the relative contribution of anaerobic glycolysis to sustain force production, thereby promoting the accumulation of lactate, hydrogen ions, and other metabolic by-products (16, 18). The resulting intramuscular acidosis has been shown to impair excitation–contraction coupling and cross-bridge cycling efficiency, accelerating the development of peripheral fatigue and contributing to decrements in force and velocity output (11, 13). Although metabolic variables were not directly assessed and no significant protocol effects were detected, this physiological framework provides a plausible explanation for the descriptive trend toward greater performance decrements observed in the 10:10 protocol compared with the longer interval configurations, despite the absence of statistically significant between-protocol differences. Instead of prescribing a fixed number of repetitions for a given load (3), the use of velocity loss as a variable to monitor resistance training volume provides a more rational and precise approach for characterizing the resistance exercise stimulus (27).

When specifically comparing the shortest and longest interval configurations, the 10:10 protocol likely imposed a greater explosive and glycolytic demand than the 30:30 protocol. The very brief work-to-rest cycles require repeated high-intent concentric actions with minimal recovery, favoring rapid phosphocreatine depletion and an increased reliance on anaerobic glycolysis to maintain power output. This metabolic profile promotes the accumulation of lactate and hydrogen ions, which are known to impair excitation–contraction coupling and accelerate peripheral fatigue (11, 13). In contrast, the longer work and rest durations of the 30:30 protocol provide a greater opportunity for partial phosphocreatine resynthesis and metabolite clearance between bouts, allowing a relatively higher aerobic contribution to energy resynthesis and a more stable maintenance of movement velocity across the session (16, 18). This differential balance between glycolytic stress and recovery availability offers a physiological explanation for the greater fatigue tendency observed in the 10:10 protocol, despite similar overall magnitudes of neuromuscular fatigue across conditions.

This study implemented a 1:1 work-to-rest ratio across three protocols, emphasizing time as the primary variable. Exercise volume was assessed using the conventional approach, defined as the total number of repetitions performed per set (3, 31, 32), regardless of the time and velocity of each repetition (33). However, the concept of pace—the cadence at which each repetition is executed—also plays a crucial role (29, 33, 34). Our results revealed that, despite the same work-to-rest ratio, differences in the number of repetitions performed across protocols suggested that fatigue influenced the pace of execution, leading to variations in movement velocity during specific phases. In particular, comparison of the 10:10, 20:20, and 30:30 protocols revealed that longer rest intervals (30 s) enabled participants to maintain a steadier number of repetitions per exercise (22, 35, 36). This finding supports previous postulations that the appropriate duration of rest interval may depend on the duration of work interval itself (22) a consideration that was prioritized in the present study rather than focusing exclusively on the number of repetitions completed during the pre-established work period. Moreover, prior literature has demonstrated that even with markedly lower repetitions, deliberately slower movement paces can induce greater postexercise fatigue and a more pronounced decline in muscle power compared with protocols performed at volitional speeds (37).

Several limitations of the present study should be acknowledged. Although a randomized and counterbalanced design was used, the acute nature of the investigation does not allow extrapolation of the observed responses to long-term neuromuscular or metabolic adaptations. Therefore, practical recommendations derived from these findings should be interpreted with caution. Metabolic variables such as oxygen uptake or phosphocreatine resynthesis were not directly assessed. As a result, the proposed physiological mechanisms underlying the observed fatigue patterns were inferred from established literature rather than directly measured responses. Finally, although training loads were individualized and velocity-based measures were employed to enhance internal validity, controlling relative intensity across different time-constrained protocols remains challenging in circuit training settings, which may have introduced variability in the actual stimulus experienced by participants.

Future research should aim to replicate these findings using chronic training interventions to determine whether different work-to-rest interval configurations lead to divergent long-term adaptations in neuromuscular performance, metabolic capacity, and cardiovascular function. The inclusion of direct physiological measurements such as muscle oxygenation or phosphocreatine recovery would provide greater mechanistic insight into the fatigue responses associated with different interval structures. Moreover, future studies should explore a wider range of work-to-rest ratios, pacing strategies, and intensity prescriptions to better inform evidence-based programming of circuit training across diverse populations and training goals.

From an applied perspective, the acute neuromuscular responses observed in this study suggest that circuit training protocols with different work-to-rest interval configurations may have potential relevance within postactivation potentiation (PAP) or postactivation performance enhancement (PAPE) frameworks. The expression of PAP/PAPE depends on the balance between potentiation mechanisms and the concurrent development of fatigue, which is strongly influenced by recovery duration (38). In this context, the shorter 10:10 protocol, characterized by high explosive demands and limited recovery, tended to elicit greater acute fatigue, which may reduce its suitability as a potentiation stimulus when applied immediately before explosive performance tasks. In contrast, the longer 30:30 protocol allowed greater repetition output and partial recovery between bouts, potentially creating more favorable conditions for potentiation to emerge once fatigue subsides. Accordingly, practitioners may consider longer work-to-rest configurations when integrating circuit-based resistance exercises into warm-up routines or complex training models aimed at enhancing subsequent performance. These applications should be interpreted cautiously, as the present findings are based on acute responses and postactivation performance outcomes were not directly assessed.

## 5 Conclusions

All three protocols induced a significant decrease in MPV during both BP and SQ exercises, indicating the presence of acute neuromuscular fatigue following circuit training. However, no statistically significant differences were observed between protocols in the magnitude of MPV reduction, suggesting that fatigue levels were comparable across the different work-to-rest configurations. At the same time, the 30:30 protocol allowed a greater total number of repetitions, indicating that longer work-to-rest intervals facilitate sustained performance without reducing the overall magnitude of neuromuscular fatigue. From a practical perspective, very brief intervals such as the 10:10 protocol, especially under high loads, appear to induce greater acute fatigue and may be useful when the aim is to elicit simultaneous cardiovascular and metabolic adaptations, whereas longer intervals like 30:30 are more effective for developing or maintaining maximal strength. Overall, these findings highlight work-to-rest duration as a key variable in the acute neuromuscular response to CT, as it modulates relative intensity, training volume, and fatigue, although caution is warranted when generalizing, given the methodological constraints. For future research, there is a need to explore pace control, broader rest durations, and chronic adaptations under time- vs. repetitions-based prescriptions.

## Data Availability

The raw data supporting the conclusions of this article will be made available by the authors, without undue reservation.
